# Construction of Fusion Protein for Enhanced Small RNA Loading to Extracellular Vesicles

**DOI:** 10.3390/genes14020261

**Published:** 2023-01-19

**Authors:** Masoumeh Es-Haghi, Olga Neustroeva, Iftekhar Chowdhury, Pia Laitinen, Mari-Anna Väänänen, Nea Korvenlaita, Tarja Malm, Mikko P. Turunen, Tiia A. Turunen

**Affiliations:** A.I. Virtanen Institute for Molecular Sciences, University of Eastern Finland, Yliopistonranta 1E, 70210 Kuopio, Finland

**Keywords:** extracellular vesicles, engineered exosome, fusion protein, miRNA, shRNA, siRNA, CD9, AGO2

## Abstract

Extracellular vesicles (EVs) naturally carry cargo from producer cells, such as RNA and protein, and can transfer these messengers to other cells and tissue. This ability provides an interesting opportunity for using EVs as delivery vehicles for therapeutic agents, such as for gene therapy. However, endogenous loading of cargo, such as microRNAs (miRNAs), is not very efficient as the copy number of miRNAs per EV is quite low. Therefore, new methods and tools to enhance the loading of small RNAs is required. In the current study, we developed fusion protein of EV membrane protein CD9 and RNA-binding protein AGO2 (hCD9.hAGO2). We show that the EVs engineered with hCD9.hAGO2 contain significantly higher levels of miRNA or shRNA (miR-466c or shRNA-451, respectively) compared to EVs that are isolated from cells that only overexpress the desired miRNA or shRNA. These hCD9.hAGO2 engineered EVs also transfer their RNA cargo to recipient cells more efficiently. We were not able to detect changes in gene expression levels in recipient cells after the EV treatments, but we show that the cell viability of HUVECs was increased after hCD9.hAGO2 EV treatments. This technical study characterizes the hCD9.hAGO2 fusion protein for the future development of enhanced RNA loading to EVs.

## 1. Introduction

Small RNAs, such as microRNAs (miRNAs), are important regulators of gene expression. Small interfering RNAs (siRNAs) and small hairpin RNAs (shRNAs) are also widely used tools for biomedicine research and all of these are also currently under development for therapeutic uses in clinics. Although different liposomal carriers and other transfection reagents are useful in the research lab, they are often not sufficient for the in vivo delivery of small RNAs to correct tissue. Over the past decade, cell-originated nanoparticles, extracellular vesicles (EV), have been introduced as a more biocompatible and biological vehicle compared to the other nano-based gene delivery particles. Briefly, in a multicellular organism, intercellular communication and message delivery occur by bilayered lipid vesicles that are naturally released from the cell membrane [1]. The term EV describes a membrane-bound particle with a diameter of 40 to 1000 nm and subdivisions of EVs such as exosomes (40–100 nm) are termed according to their size and biogenesis [2]. EV carries genetic cargo (e.g., DNA, mRNA, ncRNA, and proteins) as a part of genetic language during cell-cell crosstalk. Loading of desired nucleic acid into the EVs can be carried out with a variety of methods, either by directly affecting the isolated EVs or modifying the producer cell line to overexpress the desired nucleic acid [3,4]. EV engineering provides options for both enhanced cargo loading as well as modifying the EV surface with targeting moieties. Desired nucleic acids, such as miRNAs, can be also enriched into the EVs by a constructed fusion recombinant protein, where a hybrid of EV-packaging domain and miRNA-binding domain is created to enhance the miRNA levels in EVs. Exosome membrane proteins, membrane trafficking proteins or vesicular biogenesis proteins can be applied as a platform for packaging the chosen miRNA binding protein into the exosome [5,6]. For example, Li et al. fused the tetraspanin protein CD9 to RNA-binding protein HuR to increase loading of miRNAs into EVs [7]. The future of EVs as a therapeutic RNA delivery system depends on the development of several factors: firstly, loading of therapeutic RNA should be increased to maximize the levels of active ingredient in the medicine; secondly, targeting the EVs to the target tissue should be enhanced so that the therapeutic RNA can reach the cells where it is needed; and thirdly, internalization of the EVs and the downstream functionality of the therapeutic RNA should be understood in more detail to enhance the proper actions of RNA in the cells.

The purpose of this study was to enhance the loading of miRNAs and similar small RNAs into EVs. Some strategies for loading and modifying the EV-cargo have been identified previously. These include electroporation, freeze-thaw cycles, saponin-mediated loading, and hypotonic dialysis, which have all been studied for use in exogenously loading EVs [3,4,8,9]. However, these are often not so efficient and, in particular, they are not easy to control and validate for the reliable repetition of experiments. Therefore, engineering the EVs themselves to carry more miRNA is an attractive choice. In this technical study, our preliminary effort was focused on constructing a universal miRNA loading system with high efficiency that could be applied to different producer cell types. Our constructed vector hCD9.hAGO2 expresses an EV membrane recombinant protein related to the exosome biogenesis (CD9 domain) with a universal miRNA-high binding domain (AGO2 domain). To facilitate the ease of use, the fusion recombinant protein was constructed into a lentivirus backbone plasmid with lentiviral particle-producing ability and with a sustained yield promoter. We show that the hCD9.hAGO2 fusion protein significantly enhances miRNA or shRNA packaging into EVs and therefore leads to increased uptake of RNA in the recipient cells. 

## 2. Materials and Methods

### 2.1. Materials

For generation of the fusion protein, mCLOVER-NLS-AGO2 plasmid (gifted by Dr. Markus Hafner, Laboratory of Muscle Stem Cells and Gene Regulation, National Institute of Arthritis and Musculoskeletal and Skin Diseases, National Institutes of Health, Bethesda, MD, USA) was used. Other plasmids used include the backbone pLenti-hPGK plasmid, miR-466c expressing plasmid and shRNA-451 plasmid, which have been published previously [10,11]. 

### 2.2. Cell Culture

Human embryonic kidney cell line HEK293T (ATCC: CRL-11268), mouse yolk-sac endothelial cell line C166 (ATCC: ATCC CRL-2581) and human retinal pigment epithelial cell line ARPE19 cells (ATCC: CRL-2302) were cultured in Dulbecco’s Modified Eagle Medium (DMEM) supplemented with 10% fetal bovine serum and 1% penicillin-streptomycin. HUVECs were cultured in EBM^®^ Medium with 1x EGM BulletKit supplements (Lonza, Germany).

### 2.3. Amplification of Fragments for hCD9.hAGO2 Fusion Protein Construction

Fusion recombinant protein hCD9.hAGO2 was designed by the SnapGene software (TM1.1.3) and constructed into the pLenti-hPGK backbone (Figure S1). The protein folding and molecular weights were predicated by the online prediction software Phyre 2 (Protein Homology/analog Y Recognition Engine V 2.0) [12]. The molecular weight for hCD9.hAGO2 was 129 kDa.

Human AGO2 fragment was amplified from mCLOVER-NLS-AGO2 plasmid, gifted by Dr. Markus Hafner (Laboratory of Muscle Stem Cells and Gene Regulation). The CD9 fragment was amplified from HEK293T cell cDNA.

The cDNA was made by High-Capacity cDNA Reverse Transcription Kit (Applied Biosystems™, Thermo Fisher Scientific, Waltham, MA, USA). Table 1 shows the In-Fusion primers and touch down PCR programs for amplification of fragments. The CloneAmp HiFi PCR Premix (Takara Bio USA, San Jose, CA, USA) was used for PCR reaction and the In-Fusion^®^ HD Cloning Kit (Takara Bio USA) was used for fusing and cloning fragments according to the manufacturer’s protocols. 

### 2.4. Transfection

Plasmid transfections were carried out using Polyethyleneimine (PEI, linear, M.W. 25,000; Alfa Aesar, Haverhill, MA, USA) or TransIT-2020 Transfection Reagent (Mirus Bio, Madison, WI, USA), according to the manufacturer’s instructions. siRNA against GAPDH (GAPDH Silencer Pre-designed siRNA, Thermo Fisher) was transfected using TransIT-TKO Transfection Reagent (Mirus Bio), according to the manufacturer’s instructions.

### 2.5. EV Isolation

Cell culture medium was collected from the cells and pre-cleared by centrifugation (300× *g* for 5 min, followed with 2000× *g* for 10 min in +4 °C). CCM was filtered by a 0.2 µm filter and then concentrated using the Amicon^®^ Ultra-15 centrifugal filter device (10 KDa cut-off, Merck Millipore, Burlington, MA, USA). EVs were isolated with qEV Original Size Exclusion Chromatography (SEC) Columns (Izon Science, Lyon, France), according to the manufacturer’s protocol. Fractions 2–4 were pooled as an EV sample unless otherwise stated.

### 2.6. Western Blot

The vector expression and presence of recombinant protein in the cells and secreted EVs was confirmed by Western blotting.

#### 2.6.1. Sample Preparation for Cells

HEK293T cells were transiently transfected by constructed vectors using PEI. Then, 48 h after transfection, the condition media were removed and cells were frozen. Frozen cell pellets were lysed with 300 µL RIPA lysis buffer (100 mM Tris-base, 2% triton -X100, 1% sodium deoxycholate, 0.2% sodium dodecyl sulfate, 300 mM NaCl, pH 7.4) and incubated 20 min on ice. During ice incubation, samples were vortexed and sonicated three times for 10 s (Branson 2510 Ultrasonic Cleaner). The supernatants (protein) were collected by centrifugation at 14,000× *g* for 20 min in 4 °C. Then, 48 µL of Lysates were boiled in 12 µL of sample buffer (5X) at 95 °C for 5 min and incubated on ice for SDS page. 

#### 2.6.2. Sample Preparation for EVs

For validation of fusion protein packaging in the EVs, 200 µL purified EVs was precipitated by adding 200 µL of water, 400 µL of methanol and 100 µL of chloroform. The sample solution was vortexed and centrifuged 14,000× *g* for 5 min at room temperature (RT). After discarding the top aqueous layer, the organic phase was washed with 400 µL of methanol and was centrifuged again for protein precipitation. The pellet was air-dried, resuspended and boiled in 30 µL sample buffer(1X) at 95 °C for 5 min. The boiled samples were incubated on ice for SDS page.

#### 2.6.3. SDS-PAGE and Transfer

Next, 28 µL of boiled sample was loaded on 4–15% Mini-PROTEAN TGX Precast Protein Gels (Bio-Rad Laboratories, Hercules, CA, USA) and proteins were transferred onto the PVDF membrane (Trans-Blot Turbo Mini PVDF Transfer Packs, Bio-Rad Laboratories) by Bio-Rad Trans-Blot Turbo transfer system with default program 8–9 V, 10 A for 30 min. 

#### 2.6.4. Antibody Staining and Imaging

The fusion proteins were stained with anti-human CD9 antibody (produced in mouse, 60232-1-lg, Proteintech, Rosemont, IL, USA), or anti-human AGO2 antibody (produced in rabbit, ab226943, Abcam, Boston, MA, USA). 

The membrane was incubated with 1:1000 diluted primary antibody overnight at 4 °C and then with 1:500 diluted horseradish peroxidase conjugated goat anti-mouse secondary antibody (7076, Cell Signaling Technology, Danvers, MA, USA) and goat anti-rabbit secondary antibody (7074, Cell Signaling Technology) for 2 h at room temperature. Antibody dilutions were made in Immobilon^®^ Block—CH solution (Chemiluminescent Blocker, Merck Millipore, Darmstadt, Germany). After each incubation step, membranes were washed three times in the PBS containing 0.2% Tween-20 for 5 min. Protein bands were detected using SuperSignal™ West Pico PLUS Chemiluminescent Substrate (Thermo Fisher Scientific™) with ChemiDoc XRS System (Bio-Rad Laboratories). 

### 2.7. Double Immunofluorescence Staining and Confocal Laser Scanning Imaging

The double immunofluorescence staining method was used for tracking fused proteins hCD9 and hAGO2 simultaneously. HEK293T cells were cultured on the coverslip in the 6-well plate and constructed vectors transiently transfected via TransIT-2020 Transfection Reagent. Then, 48 h after transfection, at 70–80% confluency, cells were rinsed by PBS to remove the dead cells and debris and fixed with paraformaldehyde (4% PFA in PBS) for 15 min at RT. Cells were washed three times with PBS. Permeabilization was carried out with 0.25% Triton X-100 for 15 min at RT and cells were rinsed three times for 5 min with PBS containing 0.05% Tween-20 (PBST). Unspecific binding sites were blocked with 10% normal goat serum (NGS; Merck Millipore) in PBST for 30 min with gentle shaking in RT. 

The primary and secondary antibodies were diluted In staining buffer (5% NGS in PBST). For the simultaneous double staining of fused proteins, two primary antibodies (primary anti-CD9 and primary anti-AGO2) and two secondary antibodies (Alexa Fluor-555 and Alexa Fluor-488) were diluted in the same tube and mixed. The cells were incubated with primary antibodies for 2 h in RT by gently inverting, then washed three times with PBS for 5 min in RT. For secondary staining, the Alexa Fluor-555 (anti-rabbit, 4413S, Cell Signaling Technology) and Alexa Fluor-488 (Goat anti-mouse, 4408S, Cell Signaling Technology) were used. The cells were incubated with secondary antibodies for 1.5 h by gentle shaking in RT and after three times washing with PBS, the coverslips were transferred to the microscope slide with 50 µL of mounting medium with DAPI (Vector Laboratories, Newark, CA, USA) and were sealed with nail polish. 

Confocal laser scanning microscopy was performed using a LSM700 Laser Scanning Confocal Microscope (LSM 700; Carl Zeiss Microscopy GmbH). Microscope configuration was the following: The objective lens; Plan-Apochromat 63X/1.40 oil M27. The sequential scanning with stack mode (0.5 µm for Z-scaling and 20.50 µm for stack size). The excitation: 405 nm (Blue: DAPI), 488 nm (Green: Alexa Fluor-488) and 555 nm (Red: Alexa Fluor-555).

### 2.8. Nanoparticle Tracking Analysis (NTA)

Particle number (concentration) and size distribution of collected EV samples were analyzed with NTA Nanosight NS300 (Malvern Panalytical) equipped with Blue405 laser and sCMOS camera according to the manufacturer’s instructions. Samples were diluted 200–400-fold with filtered PBS (0.2 um). For each sample, four videos of 40 s were captured at 22–25 °C with the camera level 15. Data were analyzed using detection threshold 5. An analysis was performed by instrument software (Nanosight 3.2 Dev Build 3.2.16)

### 2.9. Cryogenic Electron Microscopy (Cryo-EM)

HEK293T cells transfected with miR-466c plasmid and either with GFP plasmid (ctrl EVs) or hCD9.hAGO2 fusion protein plasmid (hCD9.hAGO2 EVs) using PEI reagent. EVs were isolated from cell culture media with qEV columns and fractions 2–4 were pooled and concentrated with Amicon MWCO 10 kDa filter units (Merck Millipore). EVs for analysis were in PBS buffer with the concentration of 1.91 × 10^10^ particles/mL as measured with NTA. EV samples were sent to Helsinki HiLIFE Cryo-EM core for imaging.

### 2.10. RNA Isolation

For the analysis of RNA loading into EVs and transfer to recipient cells, mmu-miR-466c, shRNA-451 or siRNA against GAPDH were transfected to HEK293T cells together with a fusion protein plasmid hCD9.hAGO2 or GFP plasmid as a control. Cells were seeded in density 4–4.5 × 10^6^ cells in 15 cm plate and co-transfected with 15 µg fusion protein or GFP plasmid and 15 µg miRNA or shRNA expression plasmid with 45 µg PEI reagent. For EV collection, cells were cultured in DMEM supplemented with 2% FBS. Then, 4 h after transfection, the media was changed to a fresh complete media. Following 48 h after transfection, the condition media (17 mL per plate) and EVs were isolated as previously described. 

The total RNA from purified EVs was isolated by TRIzol (TRIzol^®^ reagent; Invitrogen, Thermo Fisher Scientific) according to the manufacturer’s instructions. Then, 750 µL TRIzol reagent was used for a maximum of 200 µL EVs sample. To increase the efficiency of RNA precipitation, 1 µL GlycoBlue (Invitrogen™, Thermo Fisher Scientific) was added to the lysis sample and incubated at 80 °C for 5 min. The RNA was precipitated with 500 µL isopropanol and incubated at −20 °C overnight. For cell samples, cells were lysed directly on the plate with TRIzol and RNA isolated according to the manufacturer’s instructions. The RNA pellet was dissolved to RNase free water and used for cDNA synthesis.

### 2.11. cDNA Synthesis and RT-qPCR

For small RNA analysis, cDNA synthesis was performed using the TaqMan MicroRNA Reverse Transcription Kit (Thermo Fisher Scientific) according to the manufacturer’s protocol and analyzed with qPCR using specific TaqMan assays (mmu-miR-466c-3p ID: 464896_mat; mmu-miR-466c-5p ID: 463771_mat, hsa-miR-223 ID: PN4427975, shRNA-451 sense custom assay ID: CS70LFG; shRNA-451 antisense custom assay ID: CSRR86K; Thermo Fisher Scientific). For gene expression analysis, cDNA was synthesized using RevertAid Reverse Transcriptase (Thermo Fisher Scientific) and random hexamer primer (Thermo Fisher Scientific). cDNA quantification was performed using TaqMan Gene Expression Assays (Vegfa ID: Mm00437306_m1; Gapdh ID: Mm99999915_g1; VEGFA ID: Hs00173626_m1; GAPDH ID: 435265, Thermo Fisher Scientific). Samples were quantified by using Maxima Probe/ROX qPCR Master Mix (2X) (Thermo Fisher Scientific). Thermal cycling was performed using a LightCycler^®^ 480 Instrument II (Roche Diagnostics, Rotkreuz, Switzerland) with the following program: 10 min at 95 °C, followed by 50 cycles of 15 s at 95 °C and 60 s at 60 °C. miRNA RT-qPCR was started with an additional step of 2 min at 50 °C. RT-qPCR data were analyzed using the ΔΔCq method where normalization was available. 

### 2.12. Proteinase K and RNase A Treatments

RNase A and Proteinase K treatments were carried out to remove probable free EVs-RNA and free-EVs RNA binding proteins. The cycle threshold (Ct) values from enzyme-treated EVs were compared to no treatment groups. For this purpose, the same number of engineered exosomes per group was incubated with Proteinase K (Thermo Fisher, EO0491) at 37 °C for 30 min with 100 µg/mL final concentration, followed by incubation with RNase A (Thermo Fisher, EN0531) at 37 °C for 15 min with10 µg/mL final concentration. After RNase treatment, the samples were frozen in liquid nitrogen, quickly incubated for 10 min to inactivate the RNase enzyme and then TRIzol reagent was added to the samples.

### 2.13. Protein Concentration Analysis

Protein concentration of EV fractions was measured with the Qubit protein assay kit (Q33211; Invitrogen, Thermo Fisher Scientific) according to the manufacturer’s instructions.

### 2.14. MTT Assay 

HUVECs were seeded with a seeding density 0.01 × 10^6^ in each well of a 96-well plate at 37 °C in 5% CO2 atmosphere. The day after seeding, 10^4^ EVs per cell were added to the cells. Then, 48 h after EVs incubation, the cells were used for MTT assay according to the manufacturer’s protocol of the MTT cell viability assay kit (Biotium, Inc., Fremont, CA, USA). 

### 2.15. Statistical Analysis

Two-tailed Student’s *t*-test was used for statistical analysis when applicable. *p*-value ≤ 0.05 was considered as significant. All data are presented as means ± SD.

## 3. Results

### 3.1. Construction of the hCD9.hAGO2 Fusion Protein

**Figure 1:**
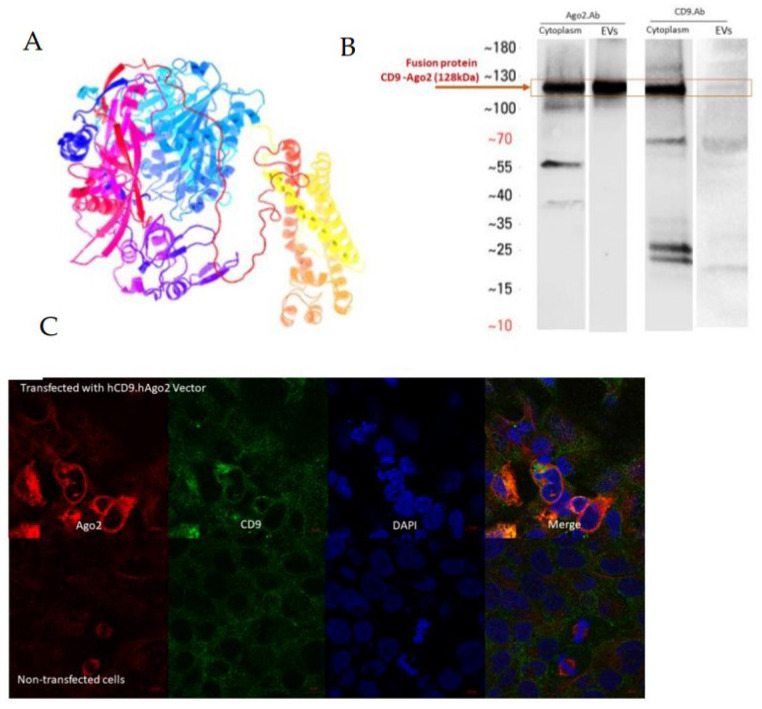

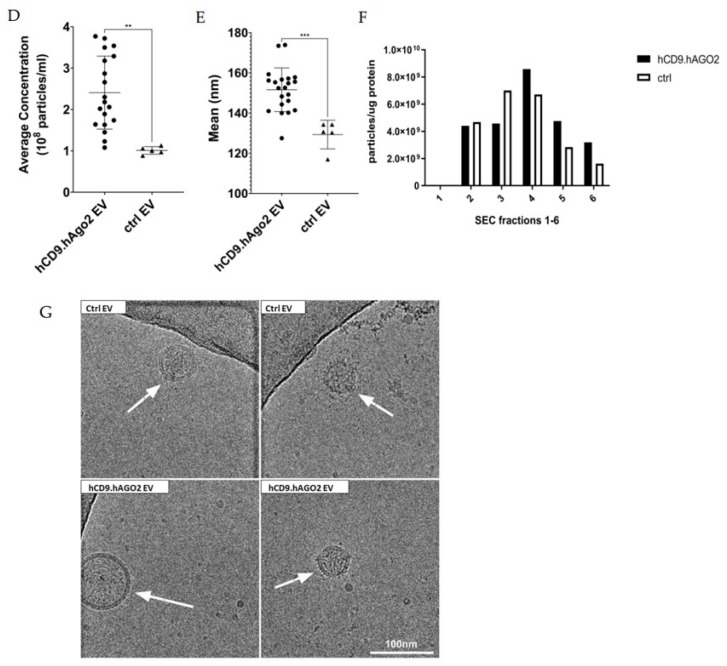
Construction of the fusion protein hCD9.hAGO2. (**A**) The protein folding by the online prediction software (Phyre.2) for hCD9.hAGO2 protein. (**B**) Western blot for hCD9.hAGO2 fusion protein after transfection to HEK293T cells (48 h timepoint) confirmed the correct fusion protein formation. Predicted molecular weight of 129 kDa was observed both in the cells and in the EVs. Unmodified gel images of western blot are supplemented in Figure S2. (**C**) Cellular colocalization of fusion protein domains was studied in HEK293T cells (24 h timepoint). The presence of recombinant hCD9.hAGO2 was confirmed by confocal microscopy after double immunofluorescence staining with CD9 and AGO2 antibodies. (**D**) Concentration of EVs in hCD9.hAGO2 EVs larger than in control groups (EVs from unmodified HEK293T cells). Measurement was made with Nanosight NS300 Nanopraticle tracking analysis (NTA). Data are presented as mean ± SD. hCD9.hAgo2 EV vs. ctrl EV *p*-value 0.0021 (**E**) Mean size of hCD9.hAGO2 EVs were determined by NTA and were observed to be statistically larger compared to the control EV group. Data are presented as mean ± SD. hCD9.hAgo2 EV vs. ctrl EV *p*-value 0.0002 (**F**) Protein concentration from different fractions of SEC EV isolation was measured and particle number to protein amount ratio was calculated for different fractions in both hCD9.hAGO2 EVs and control EVs. According to the manufacturer’s protocol (Izon Bio), fractions 2–4 have the highest amount of EVs and less contamination of proteins. We observed that compared to control EVs, hCD9.hAGO2 EVs have the highest particles to protein ratio later, at fraction 4. This may be due to the observed increased size of the EV population after hCD9.hAGO2 expression. (**G**) Cryo-EM of hCD9.hAgo2-engineered EVs. Two representative images are shown for control EVs (top row) and hCD9.hAGO2 expressing EVs (bottom row). White arrows indicate the EVs in the samples. We observed no differences in EV structure with FP loading. ** *p*-value < 0.005, *** *p*-value < 0.0005.

To enhance the small RNA loading into EVs, we fused together the EV membrane binding domain of CD9 and the RNA binding domain of AGO2 protein. CD9 is a tetraspanin family protein bound to the EV membrane with four transmembrane regions and is a commonly used EV marker protein [13]. AGO2, a member of the RNA-induced silencing complex (RISC), is important for the functionality of miRNAs, as it is able to both bind the miRNA and cleave the targeted RNA transcript for miRNA-regulated downregulation of gene expression [14]. Fusion recombinant protein hCD9.hAGO2 was designed by the SnapGene software (TM1.1.3) and cloned into the pLenti-hPGK backbone. The protein folding (Figure 1A) and molecular weight of 129 kDa was predicated by the online prediction software Phyre 2 (Protein Homology/analog Y Recognition Engine V 2.0) [12].

The expression of recombinant proteins was confirmed both in the cells (HEK293T producer cell line) and in isolated EVs using western blot. HEK293T cells were transfected with the plasmid encoding the hCD9.hAGO2 fusion protein and EVs were isolated from the cell culture supernatant using qEV size exclusion chromatography (SEC) columns from Izon Science. For hCD9.hAGO2, a molecular weight of 129 kDa was observed with antibody against AGO2 and CD9 (Figure 1B). For the C1293T cells, we also observed co-localized expression of CD9 and AGO2 using double fluorescence staining of cells (Figure 1C). The merge pictures with DAPI also showed the presence of endogenous AGO2. These results indicate the correct formation of the fusion protein hCD9.hAGO2, as well as incorporation to the secreted EVs. 

### 3.2. hCD9.hAGO2 Engineered EVs Show Difference in Mean Size of Particles and Concentration

The engineered EVs expressing hCD9.hAGO2 fusion protein were characterized using nanoparticle tracking analysis (NTA). The result showed that the concentration (Figure 1D) and mean size (Figure 1E) of hCD9.hAGO2 EVs were significantly larger than the control EV group that was isolated from unmodified HEK293T cells. The particle amount increased 2.3-fold on average after overexpressing hCD9.hAGO2 fusion protein in the producer cell line. Interestingly, CD9 overexpression has also been previously associated with increased secretion of EVs, consistent with our result [15]. From the EV isolation with the SEC method, we also analyzed the particle to protein amount ration to see the purity of different fractions (Figure 1F). The manufacturer’s (Izon Science, Lyon, France) instructions for SEC isolation are that the EVs are found in the fractions 2–4 and these fractions are less contaminated with protein. For hCD9.hAGO2 EVs, we observed the best purity in the fraction 4 (the highest number of particles per µg of protein). As the mean size of hCD9.hAGO2 EVs was observed to be increased (NTA data, Figure 1E), it may be that these EVs are separated in SEC in later fractions, as we noticed that for the non-modified EVs, the purest fractions were both 3 and 4 (Figure 1F). Since we wanted to keep the isolation procedure consistent within the experiments and treatment groups, we continued to follow the column instructions and used the pooled fractions 2–4 as the EV sample in all experiments, unless otherwise stated.

In addition, we analyzed EVs with Cryogenic Electron Microscopy (Cryo-EM) to visualize the effects of engineering on the EV structure (Figure 1G). We compared non-modified EVs produced in HEK293T cells to the engineered hCD9.hAGO2 EVs and observed no differences in EV membrane structure, indicating that the addition of the fusion protein to the EV membrane does not interfere with the normal formation of EVs and the EV structure is still intact.

### 3.3. hCD9.hAGO2 Fusion Protein Expression Significantly Enhances Small RNA Loading into the Secreted EVs

**Figure 2:**
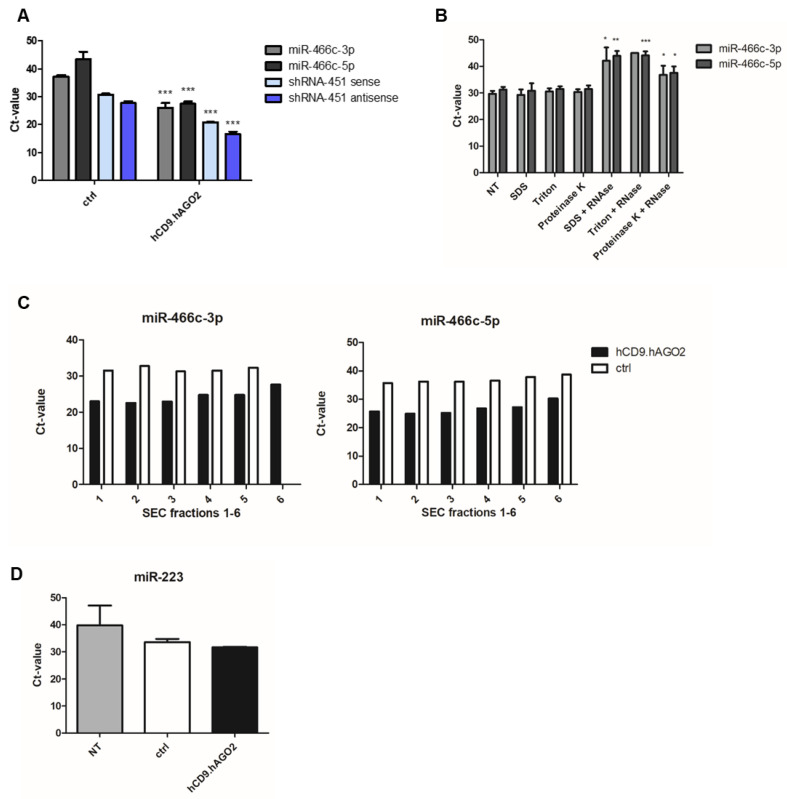
RNA-loading to EVs using the fusion protein hCD9.hAGO2. (**A**) The capability of hCD9.hAGO2 fusion protein in loading of both miR-466c and shRNA-451 in the secreted EVs was significantly increased compared to the control EVs. Data are presented as mean ± SD, *n* = 3. Two-tailed Student’s *t*-test: miR-466c-3p ctrl vs. hCD9.hAGO2 *p*-value 0.0005; miR-466c-5p ctrl vs. hCD9.hAGO2 *p*-value 0.0006; shRNA-451 sense ctrl vs. hCD9.hAGO2 *p*-value < 0.0001; shRNA-451 antisense ctrl vs. hCD9.hAGO2 *p*-value < 0.0001; (**B**) EVs loaded with miR-466c using hCD9.hAGO2 were treated with membrane disrupting agents (SDS, Triton) and protein degradation enzyme (Proteinase K) in combination with RNase A in order to see whether the miRNA is packed inside of the EVs (protected by the membrane) or outside of the EVs (in contaminating protein complexes). SDS and Triton treatments combined with RNase A caused total degradation of miR-466c as expected. Proteinase K treatment followed by RNase A did not result in as much degradation, suggesting that miR-466c is protected inside the EVs. Data are presented as mean ± SD, *n* = 3. Two-tailed Student’s *t*-test: miR-466c-3p SDS vs. SDS + RNase A *p*-value 0.0149; miR-466c-3p Proteinase K vs. Proteinase K + RNase A *p*-value 0.0352; miR-466c-5p SDS vs. SDS + RNase A *p*-value 0.0025; miR-466c-5p Triton vs. Triton + RNase A *p*-value 0.0003; miR-466c-5p Proteinase K vs. Proteinase K + RNase A *p*-value 0.0192; (**C**) From the SEC EV isolation fractions the earlier fractions (1–3) contain more miR-466c and the levels decrease starting from the fraction 4. This was seen more clearly in the hCD9.hAGO2 engineered EVs, as the levels of miR-466c are higher in the fusion protein loaded EVs than in control EVs with endogenous loading only; (**D**) After loading miR-466c to EVs either using hCD9.hAGO2 fusion protein or endogenous loading (control), we compared the expression of hsa-miR-223 between the groups and non-treated HEK293T-derived EVs (NT). hCD9.hAGO2 loading also increased the loading of endogenously expressed hsa-miR-223 even together with miR-466c overexpression in the cells. Data are presented as mean ± SD, *n* = 2. * *p*-value < 0.05, ** *p*-value < 0.005, *** *p*-value < 0.0005.

Next, we analyzed the RNA loading capacity of the engineered hCD9.hAGO2 EVs. HEK239T cells were transfected with hCD9.hAGO2 plasmid or with a plasmid encoding only GFP as a control to identify the changes between unspecific RNA loading caused by overexpression of RNA alone from the fusion protein-enhanced loading to the EVs. Cells were also co-transfected with either artificial small RNA, shRNA-451 [11], or miRNA miR-466c [10] to analyze the loading of shRNA or miRNA into EVs. We isolated RNA from the EVs and analyzed the shRNA-451 and miR-466 levels by RT-qPCR. The cycle threshold (Ct) values for both candidate small RNAs showed that the capacity of hCD9.hAGO2 fusion protein in loading of shRNAs and miRNAs in the secreted EVs was remarkably increased compared to the control exosomes (Figure 2A). The difference in average Ct-values was over 10 cycles, indicating a very good loading capacity.

RNAs can also be attached to the surface of the EVs, as EVs have been shown to accumulate a corona around them, especially in the tissues or blood, but this may also occur to some extent in the cell culture medium [16]. Therefore, we treated the miR-466c loaded hCD9.hAGO2 EVs with different lysing agents (SDS, Triton) or Proteinase K in combination with RNase A to analyze whether the miRNA is protected by the EV membrane or otherwise associated in the surface of EVs or in contaminating protein complexes. When using SDS or Triton to disrupt the membranes before RNase A, we observed strong degradation of miR-466c from the EV sample as expected (Figure 2B). When treating the samples first with Proteinase K in order to degrade all proteins that may be outside EVs and possibly protecting the miRNA, we observed a decrease in miR-466c levels after RNase A treatment, but not as much degradation as with SDS or Triton treatments. This suggests that miR-466c is also secreted outside the EVs, but there is still a proportion of the miRNA that is inside and protected by the EV membrane. 

EV isolation with SEC columns yields different fractions, where the manufacturer states that fractions 2–4 will contain most of the EVs; we used the pooled fractions throughout the study as the EV sample. For miR-466c expression levels, we analyzed the fractions separately and noted that the fractions 1–3 contain higher levels of miRNA (Figure 2C) and the miRNA levels decrease further from fraction 4 to 6. Control EVs, which only rely on the endogenous loading of miRNA to EVs as the hCD9.hAGO2 transfection is replaced with a GFP control plasmid, show very little differences between the fractions, possibly due to sensitivity issues in RT-qPCR with low expression of miRNA. 

We also analyzed another miRNA, hsa-miR-223 expression, in the isolated EVs (Figure 2D). Analyzing the EVs from unmodified HEK293T cells, miRNA overexpression only or hCD9.hAGO2 engineered cells showed that the hCD9.hAGO2 fusion protein also increased miR-223 loading to the EVs compared to the controls (Ct 31.7 vs. 33.6, hCD9.hAGO2 and ctrl, respectively). This suggests that miRNAs are increasingly loaded to EVs in the cells expressing the fusion protein, applying also to miRNAs with basal expression levels instead of overexpression from exogenous transfection.

### 3.4. miRNA Is Transferred Efficiently to Recipient Cells When Delivered with Engineered EVs, but Regulation of Target Genes Was Not Observed

**Figure 3:**
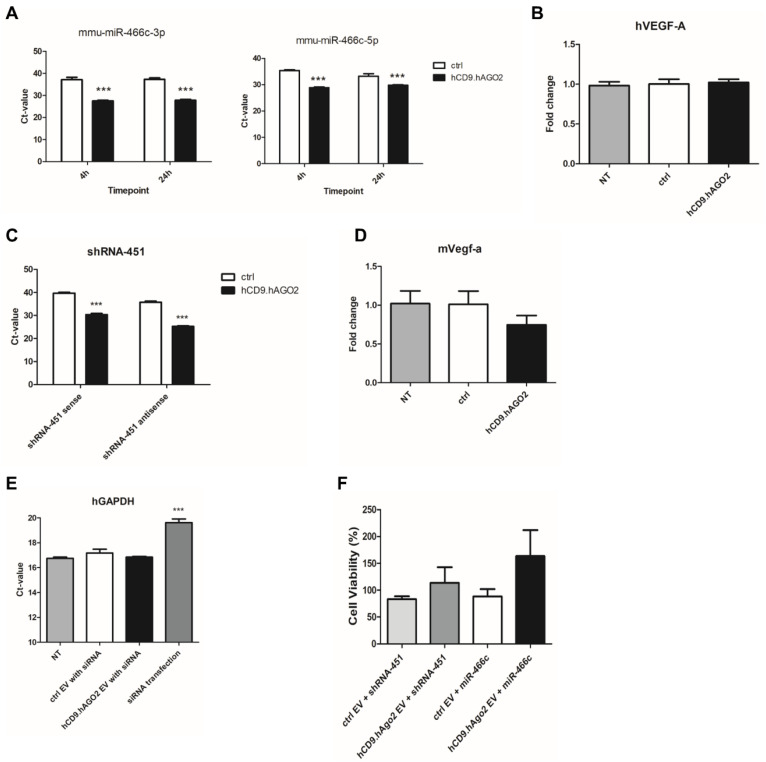
Delivery of RNA to recipient cells using hCD9.hAGO2-engineered EVs. (**A**) hCD9.hAGO2 EVs loaded with miR-466c were shown to deliver the miRNA cargo to recipient ARPE19 cell line more efficiently than control EVs (with endogenous miR-466c loading). The uptake was observed already at 4 h timepoint and also observed still at 24 h timepoint. Data are presented as mean ± SD, *n* = 4. Two-tailed Student’s *t*-test: miR-466c-3p 4 h ctrl vs. hCD9.hAGO2 *p*-value < 0.0001; miR-466c-3p 24 h ctrl vs. hCD9.hAGO2 *p*-value < 0.0001; miR-466c-5p 4 h ctrl vs. hCD9.hAGO2 *p*-value < 0.0001; miR-466c-5p 24 h ctrl vs. hCD9.hAGO2 *p*-value 0.0004; (**B**) No effect on VEGFA expression was observed in ARPE19 cells after EV-mediated delivery of miR-466c. Data are presented as mean ± SD, *n* = 3; (**C**) Transfer of shRNA-451 from the EVs to recipient C166 cell line was observed by qRT-PCR. The fusion protein-enhanced loading to hCD9.hAGO2 EVs increased the shRNA-451 amount taken up in the cells. Data are presented as mean ± SD, *n* = 3. Two-tailed Student’s *t*-test: shRNA-451 sense ctrl vs. hCD9.hAGO2 *p*-value < 0.0001; shRNA-451 antisense ctrl vs. hCD9.hAGO2 *p*-value < 0.0001; (**D**) No effect on Vegfa expression was observed in C166 cells after the EV treatments (timepoint 7 d). Data are presented as mean ± SD, *n* = 3; (**E**) siRNA against GAPDH mRNA was loaded to EVs (hCD9.hAGO2 enhanced loading or endogenous loading as a control). ARPE19 cells were treated with the EVs but no change at GAPDH expression levels was observed. As a positive control, we used normal liposomal transfection reagent to deliver the siRNA and observed clear decrease in GAPDH mRNA levels as expected. Data are presented as mean ± SD, *n* = 3. Two-tailed Student’s *t*-test: NT vs. siRNA transfection *p*-value < 0.0001; (**F**) siRNA against GAPDH mRNA was loaded to EVs (hCD9.hAGO2 enhanced loading or endogenous loading as a control). ARPE19 cells were treated with the EVs but no change at GAPDH expression levels were observed. As a positive control, we used normal liposomal transfection reagent to deliver the siRNA and observed clear decrease in GAPDH mRNA levels as expected. Data are presented as mean ± SD, *n* = 3. Two-tailed Student’s *t*-test: NT vs. siRNA transfection *p*-value < 0.0001.

To analyze whether the miRNAs loaded to the EVs are able to transfer to a recipient cell line, human ARPE19 cell line was treated with EVs for 4 h or 24 h (Figure 3A). Uptake of miRNA was measured by RT-qPCR for both arms of mature miR-466c, miR-466c-3p and miR-466c-5p. Uptake in recipient cells increased when miR-466c was loaded with the hCD9.hAGO2 fusion protein to the EVs, although EVs that had been collected from HEK293T cells overexpressing only miR-466c (endogenous loading without fusion protein, ctrl) showed some transfer of the miRNA to recipient cells as well. As we have previously shown that miR-466c induces Vegf-a expression by the promoter targeting mechanisms [10], we also analyzed if the EV treatment induces changes in the gene expression (Figure 3B). However, no changes in VEGF-A levels were observed in either the control group (endogenous loading) or the hCD9.hAGO2 engineered EV group. 

In addition to miR-466c, we loaded shRNA-451 to EVs to study the effect on the recipient cell. We previously published this shRNA as a regulator of Vegfa gene expression in mice [11,17]. As with miR-466c (Figure 3A), we observed by RT-qPCR efficient uptake in a mouse endothelial cell line C166 (Figure 3C). However, also in this case, we did not detect any changes in Vegfa expression (Figure 3D). 

As we speculated that the limited changes observed in the gene regulation may be due to inadequate transfer of miRNA or shRNA to the nucleus, we also loaded siRNA against the GAPDH gene into EVs with either fusion protein hCD9.hAGO2 or endogenous loading (GFP plasmid control). We compared the siRNA loaded EV treatments to traditional siRNA transfection as a positive control but were not able to detect any significant changes in either EV group (Figure 3E). It may be that the transferred siRNA amount via EVs, even with enhanced loading with hCD9.hAGO2 fusion protein, is not enough to cause detectable changes in this highly expressed housekeeping gene. It is also possible that siRNA is not loaded into EVs similarly to miRNAs or shRNAs with the fusion protein, or that the fusion protein-bound small RNAs have some other hindrance that inhibit their functionality in the cell.

The cell viability rate in the recipient cells was increased by miRNA-loaded hCD9.hAGO2 EVs.

We performed MTT assay to analyze whether the hCD9.hAGO2 fusion protein EVs would have impact on cell viability. Our previous research on shRNA-451 and miR-466c has identified these small RNAs as important regulators of Vegfa biology in endothelial cells [10,11], therefore we chose human umbilical vein endothelial cells (HUVEC) to be used as the recipient cell line in the experiment. Using HUVECs also provided more primary cell types for testing the system. In the treatments, we used EVs loaded with shRNA-451 or miR-466c, either endogenously (ctrl) or with hCD9.hAGO2 engineering. Interestingly, we noticed that the viability of cells was better in the groups that received the hCD9.hAGO2 EVs loaded with either shRNA or miRNA than in their respective control groups (Figure 3F). These results show that the fusion protein hCD9.hAGO2 EVs may exert some biological effect in recipient cells, even though the changes in VEFGA expression levels were not detectable. 

## 4. Discussion

Efficient RNA loading into EVs is important not only for research purposes but also for therapeutic applications as a medicine carrier. This can be achieved with a variety of techniques, such as physically or chemically opening the EV membrane to load cargo or relying on passive loading. It has been shown that the passive RNAs packing into EVs depends on RNA structure and membrane order, which may cause bias in the RNAs that are loaded [18]. Currently, there are some components proposed for controlling a selective sorting of RNA. For example, most of the miRNAs that are more prominently identified in EVs have a specific short motif that is recognized by heterogeneous nuclear ribonucleoprotein A2/B1 (hnRNPA2B1) as a result of sumoylation [19]. Therefore, sumoylation of hnRNPA2B1 leads to specifically binding to miR-198 and packaging into the EVs. MS2 protein is a bacteriophage coat protein dimer that possesses RNA-binding activity. Hung and Leonard demonstrated that modified RNA that included three high-affinity loops to MS2 had higher binding activity to bacteriophage proteins located on the surface of EVs [20]. Nevertheless, more studies on different active loading mechanisms and tools are still required, so that all kinds of RNAs can be loaded into all types of EVs. 

In this study, we aimed to actively aid the sorting of short RNAs, namely miRNAs, into EVs. For this purpose, we fused human AGO2, the effective unit of RISC complex binding functional miRNAs in the cells, into the EV transmembrane domain of human CD9 protein. Our rationale was that miRNAs would bind the hCD9.hAGO2 fusion protein in the producer cell efficiently, as it is overexpressed in cells at high levels and would thus compete with the endogenous binding of miRNAs to the cell’s own unmodified AGO2. The CD9 domain would then aid the sorting of the miRNA-fusion protein-complex to EVs during production in producer cells. 

As shown in this paper, this construct indeed efficiently increased the amount of miRNA in the EVs compared to only overexpressing the miRNA in producer cells and relying only on the endogenous miRNA sorting to the EVs. We show here that the miRNAs loaded into EVs with the help of the fusion protein are efficiently transferred to recipient cells. This was tested both with human cell line ARPE19 and mouse cell line C166. 

Our technical experiments demonstrated that the transfer of miRNAs and shRNAs was both successful, which we also assumed because of their structural similarities. We have previously published shRNA-451 and miR-466c in the regulation of Vegfa expression [10,11]. This regulation occurs through targeting the Vegfa gene promoter instead of the mRNA 3′UTR and leads to activation of the gene expression, rather than the decrease in Vegfa levels as in traditional post-transcriptional gene silencing (PTGS). Therefore, we assessed the functional effects of the shRNA or miRNA with EV delivery but were not able to detect effects in the recipient cells. This may be due to many reasons, some of which may be technical (incorrect timepoint of analysis) or due to the functionality of the fusion protein. We were not able to determine whether the shRNA or miRNA is able to transfer to the cell nucleus, where it should exert its effects on the Vegfa gene promoter. Since siRNAs have a more traditional role in the PTGS, we also tried loading siRNA against the GAPDH housekeeping gene to the EVs, but again were not able to see functional effects in the cell. It is possible that the experimental setup was inaccurate for detecting small changes in the gene expression levels of the housekeeping gene, but it is also possible that the siRNA is not exerting proper actions since it is bound to the fusion protein and possibly will not be able to bind functional RISC in the recipient cell. GAPDH is highly expressed in the cells as a housekeeping gene, which are cellular maintenance genes that regulate basic and ubiquitous cellular functions. Therefore, it is also possible that although we increased the siRNA transfer to the recipient cells, the amount of siRNA molecules is still not sufficient for downregulating the mRNA levels in the cells to the extent that it could be significantly detected by qPCR. Interestingly, cell viability was improved by the hCD9.hAGO2-mediated shRNA-451 or miR-466c delivery. This may imply that either the RNAs are released in the cells and can exert their functions, even though we do not detect significant changes in Vegfa expression levels, or the modified EVs themselves have an otherwise positive influence on the treated cells.

In conclusion, the hCD9.hAGO2 fusion protein constructed for this study shows great potential with enhanced small RNA loading to EVs. More work is still required to analyze and understand the process it undergoes in the recipient cell and how to improve the functionality of the small RNA in the cell after the uptake. As different RNA therapies are emerging, tools for delivering the RNA efficiently to target tissue and cells are required and, as natural, non-immunogenic particles, EVs provide an alluring option for drug delivery. The next steps for enhancing endogenous EV delivery vehicles are to enhance drug loading, directing the EV uptake to the desired cell population and enhancing cargo internalization and functionality within the target cells. The study here presents one possible alternative for enhanced RNA loading with hCD9.hAGO2 fusion protein, providing a tool for delivery vehicle development.

## Figures and Tables

**Table 1:** The list of designed In-Fusion PCR primers by SnapGene and touch down PCR programs for fusion protein fragments amplification.

Cloning Project	Fragment Amplification	Designed Primers	PCR Program
hCD9.hAgo2	hCD9 (with Kozak, without stop codon)	Fwd: GCCGCCACCatgccggtcaaaggaggca Rev: gaccatctcgcggttcctg	94 °C, 5 min 14X (94 °C, 30 s/64 °C, 30 s decreased −0.5 °C each cycle/72 °C, 7 s) 19X (94 °C, 30 s/57 °C, 30 s/72 °C, 7 s)
	hCD9 (15 bp homology with LV.vector)	Fwd: agggggatccaccggGCCGCCACCatgccg Rev: gcggcggcgaccatctcgcggttcctg	94 °C, 5 min 14X (94 °C, 30 s/64.3 °C, 30 s decreased −0.5 °C each cycle/72 °C, 7 s) 19X (94 °C, 30 s/57.3 °C, 30 s/72 °C, 7 s)
	HA.FLAG.hAgo2 (15 bp homology with LV. vector and 6 bp homology with CD9 fragment)	Fwd: gatggtcgccgccgccatggac Rev: gaggttgattgtcgatcaagcaaagtacatggtgcgc	94 °C, 5 min 14X (94 °C, 30 s/63.7 °C, 30 s decreased −0.5 °C each cycle/72 °C, 14 s) 19X (94 °C, 30 s/56.7 °C, 30 s/72 °C, 14 s)

## Data Availability

The data presented in this study are available on request from the corresponding author.

## Supplementary Materials

The following supporting information can be downloaded at: https://www.mdpi.com/article/10.3390/genes14020261/s1, Figure S1: Plasmid structure of the hCD9.hAGO2 construct. Figure S2: Unmodified western blot gel images.
