# Supplementary material for: Construction of Fusion Protein for Enhanced Small RNA Loading to Extracellular Vesicles
Source: Genes (Basel). 2023 Jan 19;14(2):261. doi: 10.3390/genes14020261 (PMC9956110; doi:10.3390/genes14020261)
Supplement: Supplementary file 1 [file genes-14-00261-s001.zip › genes-2143789-supplementary.pdf]

Supplementary Figures

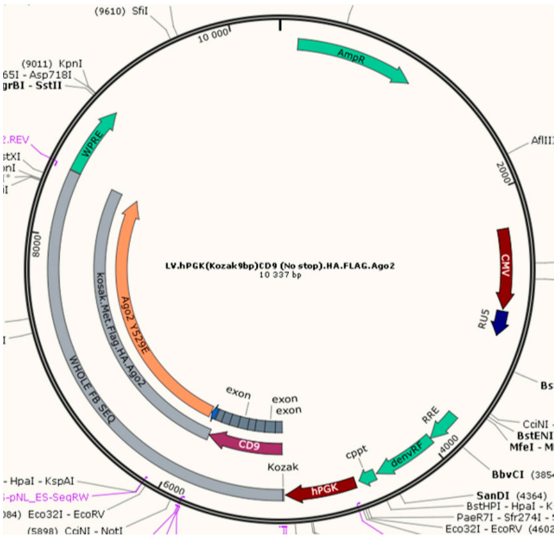

Supplementary Figure S1. Plasmid structure of the hCD9.hAGO2 construct.

Cell samples

Ago2 antibody

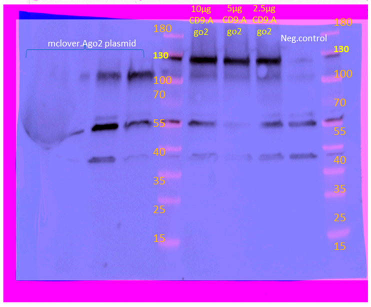

CD9 antibody

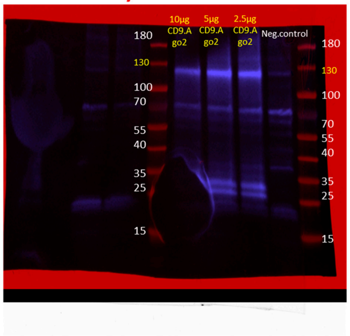

EV samples

Ago2 antibody

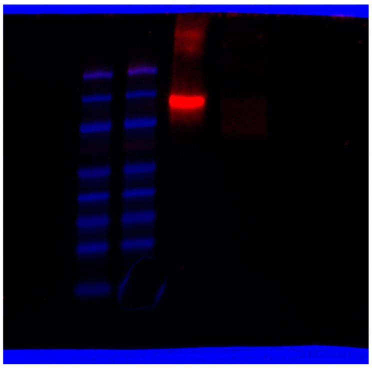

Supplementary Figure S2. Unmodified western blot gel images.
